# Noninvasive ventilation in COVID-19 patients aged ≥ 70 years—a prospective multicentre cohort study

**DOI:** 10.1186/s13054-022-04082-1

**Published:** 2022-07-22

**Authors:** Kamil Polok, Jakub Fronczek, Antonio Artigas, Hans Flaatten, Bertrand Guidet, Dylan W. De Lange, Jesper Fjølner, Susannah Leaver, Michael Beil, Sigal Sviri, Raphael Romano Bruno, Bernhard Wernly, Bernardo Bollen Pinto, Joerg C. Schefold, Dorota Studzińska, Michael Joannidis, Sandra Oeyen, Brian Marsh, Finn H. Andersen, Rui Moreno, Maurizio Cecconi, Christian Jung, Wojciech Szczeklik, COVIP Study Group, Philipp Eller, Michael Joannidis, Dieter Mesotten, Pascal Reper, Sandra Oeyen, Walter Swinnen, Helene Brix, Jens Brushoej, Maja Villefrance, Helene Korvenius Nedergaard, Anders Thais Bjerregaard, Ida Riise Balleby, Kasper Andersen, Maria Aagaard Hansen, Stine Uhrenholt, Helle Bundgaard, Jesper Fjølner, Aliae A. R. Mohamed Hussein, Rehab Salah, Yasmin Khairy NasrEldin Mohamed Ali, Kyrillos Wassim, Yumna A. Elgazzar, Samar Tharwat, Ahmed Y. Azzam, Ayman abdelmawgoad habib, Hazem Maarouf Abosheaishaa, Mohammed A. Azab, Susannah Leaver, Arnaud Galbois, Bertrand Guidet, Cyril Charron, Emmanuel Guerot, Guillaume Besch, Jean-Philippe Rigaud, Julien Maizel, Michel Djibré, Philippe Burtin, Pierre Garcon, Saad Nseir, Xavier Valette, Nica Alexandru, Nathalie Marin, Marie Vaissiere, Gaëtan Plantefeve, Thierry Vanderlinden, Igor Jurcisin, Buno Megarbane, Anais Caillard, Arnaud Valent, Marc Garnier, Sebastien Besset, Johanna Oziel, Jean-herlé RAPHALEN, Stéphane Dauger, Guillaume Dumas, Bruno Goncalves, Gaël Piton, Eberhard Barth, Ulrich Goebel, Eberhard Barth, Anselm Kunstein, Michael Schuster, Martin Welte, Matthias Lutz, Patrick Meybohm, Stephan Steiner, Tudor Poerner, Hendrik Haake, Stefan Schaller, Stefan Schaller, Stefan Schaller, Detlef Kindgen-Milles, Christian Meyer, Muhammed Kurt, Karl Friedrich Kuhn, Winfried Randerath, Jakob Wollborn, Zouhir Dindane, Hans-Joachim Kabitz, Ingo Voigt, Gonxhe Shala, Andreas Faltlhauser, Nikoletta Rovina, Zoi Aidoni, Evangelia Chrisanthopoulou, Antonios Papadogoulas, Mohan Gurjar, Ata Mahmoodpoor, Abdullah khudhur Ahmed, Brian Marsh, Ahmed Elsaka, Sigal Sviri, Vittoria Comellini, Ahmed Rabha, Hazem Ahmed, Silvio A. Namendys-Silva, Abdelilah Ghannam, Martijn Groenendijk, Marieke Zegers, Dylan de Lange, Alex Cornet, Mirjam Evers, Lenneke Haas, Tom Dormans, Willem Dieperink, Luis Romundstad, Britt Sjøbø, Finn H. Andersen, Hans Frank Strietzel, Theresa Olasveengen, Michael Hahn, Miroslaw Czuczwar, Ryszard Gawda, Jakub Klimkiewicz, Maria de Lurdes Campos Santos, André Gordinho, Henrique Santos, Rui Assis, Ana Isabel Pinho Oliveira, Mohamed Raafat Badawy, David Perez-Torres, Gemma Gomà, Mercedes Ibarz Villamayor, Angela Prado Mira, Patricia Jimeno Cubero, Susana Arias Rivera, Teresa Tomasa, David Iglesias, Eric Mayor Vázquez, Cesar Aldecoa, Aida Fernández Ferreira, Begoña Zalba-Etayo, Isabel Canas-Perez, Luis Tamayo-Lomas, Cristina Diaz-Rodriguez, Susana Sancho, Jesús Priego, Enas M. Y. Abualqumboz, Momin Majed Yousuf Hilles, Mahmoud Saleh, Nawfel Ben-HAmouda, Andrea Roberti, Alexander Dullenkopf, Yvan Fleury, Bernardo Bollen Pinto, Joerg C. Schefold, Mohammed Al-Sadawi

**Affiliations:** 1 Centre for Intensive Care and Perioperative Medicine, Jagiellonian University Medical College, ul. Wrocławska 1-3, 30 – 901 Kraków, Poland; 2 Department of Pulmonology, Jagiellonian University Medical College, Kraków, Poland; 3 Critical Care Department, Corporacion Sanitaria Universitaria Parc Tauli, CIBER Enfermedades Respiratorias, Autonomous University of Barcelona, Sabadell, Spain; 4 Department of Anaesthesia and Intensive Care, Haukeland University Hospital, Bergen, Norway; 5 Department of Clinical Medicine, University of Bergen, Bergen, Norway; 6 INSERM, UMR_S 1136, Institut Pierre Louis d’Epidémiologie et de Santé Publique, Equipe: Epidémiologie Hospitalière Qualité et Organisation des Soins, Sorbonne Universités, UPMC Univ Paris 06, 75012 Paris, France; 7 Assistance Publique - Hôpitaux de Paris, Paris, France; 8 Department of Intensive Care Medicine, University Medical Center, University Utrecht, Utrecht, The Netherlands; 9 Department of Anaesthesia and Intensive Care, Viborg Regional Hospital, Viborg, Denmark; 10 Department of Critical Care Medicine, St George’s Hospital, London, UK; 11 Medical Intensive Care Unit, Hadassah Medical Center, Jerusalem, Israel; 12 Department of Medical Intensive Care, Hadassah Medical Center and Faculty of Medicine, Hebrew University of Jerusalem, Jerusalem, Israel; 13 Department of Cardiology, Pulmonology and Vascular Medicine, Medical Faculty, Heinrich-Heine-University Duesseldorf, Moorenstraße 5, 40225 Duesseldorf, Germany; 14 Department of Internal Medicine, General Hospital Oberndorf, Teaching Hospital of the Paracelsus Medical University, Salzburg, Austria; 15 Institute of General Practice, Family Medicine and Preventive Medicine, Paracelsus Medical University of Salzburg, Salzburg, Austria; 16 Department of Acute Medicine, Geneva University Hospitals, Geneva, Switzerland; 17 Department of Intensive Care Medicine, Inselspital, Bern University Hospital, University of Bern, Bern, Switzerland; 18 Division of Intensive Care and Emergency Medicine, Department of Internal Medicine, Medical University Innsbruck, Innsbruck, Austria; 19 Department of Intensive Care 1K12IC, Ghent University Hospital, Ghent, Belgium; 20 Department of Critical Care Medicine, Mater Misericordiae University Hospital, Dublin, Ireland; 21 Department of Anaesthesia and Intensive Care, Ålesund Hospital, Ålesund, Norway; 22 Department of Circulation and Medical Imaging, Norwegian University of Science and Technology, Trondheim, Norway; 23 Faculdade de Ciências Médicas de Lisboa - Nova Médical School, Hospital de São José, Centro Hospitalar Universitário de Lisboa Central, Lisbon, Portugal; 24 Faculdade de Ciências da Saúde, Universidade da Beira Interior, Covilhã, Portugal; 25 Department of Anesthesia and Intensive Care Medicine, Humanitas Clinical and Research Center – IRCCS, Via Alessandro Manzoni 56, 20089 Rozzano, MI Italy; 26 Department of Biomedical Sciences, Humanitas University, Pieve Emanuele, Rozzano, MI Italy

**Keywords:** COVID-19, Noninvasive ventilation, Frailty, Intensive care unit, Elderly

## Abstract

**Background:**

Noninvasive ventilation (NIV) is a promising alternative to invasive mechanical ventilation (IMV) with a particular importance amidst the shortage of intensive care unit (ICU) beds during the COVID-19 pandemic. We aimed to evaluate the use of NIV in Europe and factors associated with outcomes of patients treated with NIV.

**Methods:**

This is a substudy of COVIP study—an international prospective observational study enrolling patients aged ≥ 70 years with confirmed COVID-19 treated in ICU. We enrolled patients in 156 ICUs across 15 European countries between March 2020 and April 2021.The primary endpoint was 30-day mortality.

**Results:**

Cohort included 3074 patients, most of whom were male (2197/3074, 71.4%) at the mean age of 75.7 years (SD 4.6). NIV frequency was 25.7% and varied from 1.1 to 62.0% between participating countries. Primary NIV failure, defined as need for endotracheal intubation or death within 30 days since ICU admission, occurred in 470/629 (74.7%) of patients. Factors associated with increased NIV failure risk were higher Sequential Organ Failure Assessment (SOFA) score (OR 3.73, 95% CI 2.36–5.90) and Clinical Frailty Scale (CFS) on admission (OR 1.46, 95% CI 1.06–2.00). Patients initially treated with NIV (n = 630) lived for 1.36 fewer days (95% CI − 2.27 to − 0.46 days) compared to primary IMV group (n = 1876).

**Conclusions:**

Frequency of NIV use varies across European countries. Higher severity of illness and more severe frailty were associated with a risk of NIV failure among critically ill older adults with COVID-19. Primary IMV was associated with better outcomes than primary NIV.

*Clinical Trial Registration* NCT04321265, registered 19 March 2020, https://clinicaltrials.gov.

**Supplementary Information:**

The online version contains supplementary material available at 10.1186/s13054-022-04082-1.

## Introduction

Coronavirus disease 2019 (COVID-19) led to an unprecedented disruption of everyday life and insufficiency of healthcare systems around the world [1]. As a result, tremendous efforts were made by researchers to elucidate the disease pathophysiology, find effective treatments, and develop vaccines [2–5]. COVID-19 typically involves the respiratory system and may lead to an acute respiratory distress syndrome (ARDS) with a poor prognosis and frequent need for invasive mechanical ventilation [6]. It is estimated that approximately 9% of hospitalized patients become critically ill and require transfer to an intensive care unit (ICU) [7]. Unfortunately, the sheer volume of the most severely ill patients repeatedly led to an overload of ICUs. As a consequence, the management of severe hypoxemic acute respiratory failure (ARF) required an adjustment to the reality of the global pandemic.

According to the current guidelines, the primary method of respiratory support in patients with ARDS is invasive mechanical ventilation (IMV). Conversely, noninvasive ventilation (NIV) is reserved for selected patients with mild ARDS. It should be used with caution and in constant preparedness for endotracheal intubation [8]. However, due to the shortage of available ICU beds, a significant proportion of COVID-19 patients with ARDS have been treated with noninvasive methods, including high-flow oxygen therapy and noninvasive ventilation, often outside the ICU [9–11]. This approach was based on available evidence of reduced intubation and mortality rates in patients with hypoxemic non-hypercapnic ARF treated with NIV [12].

Due to multimorbidity and frailty, critically ill elderly patients have a particularly poor prognosis [13]. Similar analyses among patients with COVID-19 confirmed that increasing age and degree of frailty are related to worse outcomes in this population [14]. Compared to NIV, endotracheal intubation and IMV are associated with more discomfort and a higher risk of complications, e.g., ventilator-associated pneumonia. Hence, NIV may be a particularly appealing therapeutic option in elderly patients with COVID-19, including those with the “do not intubate” order. The available evidence suggests that NIV is superior to high-flow nasal oxygen therapy (HFNOT) and conventional oxygen therapy in terms of decreasing 30-day intubation rate in these patients, although no effect on mortality was observed [15, 16]. To date, there are no large-scale studies describing the use of NIV among old patients in Europe and evaluating outcomes in this clinical context.

This substudy of the COVIP study aimed to describe the use of NIV in critically ill older adults with COVID-19 admitted to European ICUs. Moreover, we attempted to assess the outcomes in patients treated with NIV, identify risk factors for NIV failure, and compare the effects of primary NIV and primary IMV in this population.

## Methods

### Study design

This is a substudy of the COVIP study, an international prospective cohort study that recruited patients aged ≥ 70 years with confirmed COVID-19 admitted to the ICU. The COVIP study aims to assess outcomes and factors associated with the outcomes in the population of elderly ICU patients with COVID-19. It is a part of the Very old Intensive Care Patients (VIP) research network, which includes critical care physicians and researchers from around the world and is focused on investigating the management and outcomes of VIPs [14, 17]. Patients included in this substudy were recruited in 156 centres from 15 countries between March 2020 and April 2021. Detailed information about participating countries and number of enrolled patients is summarized in Table 1. National study coordinators were responsible for gaining local ethical permission, supervision of patient recruitment, and recruitment of ICUs. Ethical consent procedures were highly variable across participating countries (Additional file 1: Table S1). The study was performed in accordance with the Declaration of Helsinki and its amendments.

**Table 1:** Noninvasive ventilation rate and application across included countries

Country	Number of patients	Number of patients using NIV	NIV rate % (95% CI)	NIV application	
				Primary NIV	Post-extubation NIV
Austria	40	10	25.0% (12.7–41.2%)	9 (90.0)	1 (10.0)
Belgium	174	2	1.1% (0.1–4.1%)	2 (100.0)	0 (0.0)
Denmark*	215	73	34.0% (27.7–40.7%)	66 (90.4)	6 (8.2)
England	172	94	54.7% (46.9–62.2%)	89 (94.7)	5 (5.3)
France	699	170	24.3 (21.2–27.7%)	124 (72.9)	46 (27.1)
Germany*	284	141	49.6% (43.7–55.6%)	124 (87.9)	15 (10.6)
Greece	130	36	27.7% (20.2–36.2%)	19 (52.8)	17 (47.2)
Israel	58	20	34.5% (22.5–48.1%)	15 (75.0)	5 (25.0)
Netherlands	338	11	3.3% (1.6–5.8%)	7 (63.6)	4 (36.4)
Norway	23	12	52.2% (30.6–73.2%)	12 (100.0)	0 (0.0)
Poland	129	4	3.1% (0.9–7.8%)	2 (50.0)	2 (50.0)
Portugal	91	48	52.7% (42.0–63.3%)	39 (81.3)	9 (18.7)
Spain*	408	46	11.3% (8.4–14.8%)	30 (66.0)	15 (34.0)
Switzerland	263	93	35.4% (29.6–41.5%)	63 (67.7)	30 (32.3)
Wales	50	31	62.0% (47.2–75.4%)	29 (93.5)	2 (6.5)

*NIV*—noninvasive ventilation

*Day of NIV initiation was unknown in 2 patients from Germany and 1 patient from Denmark and Spain

### Study population and data collection

The COVIP study included patients admitted to the ICU and aged ≥ 70 years in whom SARS-CoV2 infection was confirmed using reverse transcription-polymerase chain reaction (RT-PCR). Should a patient be previously enrolled in the COVIP study, he or she would not be recruited again upon transfer, readmission or for any other circumstance.

Information about patients was gathered using electronic case report forms. The date of the ICU admission was labeled as day 1, and all dates were numbered sequentially from that day on. The study personnel gathered detailed clinical data on each patient, including baseline demographic and clinical characteristics (see definitions of comorbidities in Additional file 1: Table S2), Sequential Organ Failure Assessment (SOFA), and Clinical Frailty Scale (CFS) scores at admission. Based on the CFS score, patients were categorized as fit (1–3 points), vulnerable (4 points) or frail (5–9 points). We additionally gathered information about NIV (day of initiation and duration) and invasive ventilation (day of intubation and duration of IMV). Patients were included in the primary NIV group when NIV was the initial mechanical ventilation modality. At the same time, patients in whom IMV was introduced as the first respiratory support were included in the primary IMV group.

The patients were followed-up throughout the hospitalization and up to three months after admission to the ICU. The primary endpoint for this substudy was mortality within 30 days from admission to the ICU. The secondary endpoint was NIV failure defined as death or need for intubation within 30 days of admission to the ICU.

### Statistical analysis

We presented categorical variables as numbers (percentages) and we compared them using the Chi^2^ test, while continuous variables were presented as medians with interquartile ranges (IQR) and compared using the Mann–Whitney test. Comparisons of crude mortality between study groups were performed using the log-rank test and were visualized using the Kaplan–Meier curves.

Multivariable analysis of the association between pre-intubation NIV duration and 30-day mortality was performed using logistic regression. It included all-cause mortality at 30 days as dependent variable and the interval from NIV initiation to intubation in days treated as a continuous variable as well as the set of the following independent variables selected according to available literature and expert knowledge: number of days hospitalized prior to ICU admission, age, sex, body mass index (BMI), SOFA score on admission, CFS score on admission, hypertension, ischaemic heart disease [IHD], diabetes, chronic pulmonary disease, chronic kidney disease, congestive heart failure. The linear association between the pre-intubation NIV duration and 30-day mortality was later presented graphically.

Evaluation of factors associated with NIV failure was performed using logistic regression with NIV failure as a dependent variable and same set of independent variables as in the model mentioned above. We performed a sensitivity analysis for which we excluded patients in whom decision to withhold or withdraw life-sustaining treatment was made within 2 days since admission to the ICU. Finally, we assessed intubation rate and factors associated with it in a subgroup of patients from the primary NIV group in whom LST limitation was not introduced during the NIV therapy period.

To compare survival in the primary NIV and the primary IMV groups, we performed a multivariable survival analysis using restricted mean survival time adjusted for the limitation of life-sustaining therapies (LST) during the hospitalization and the above mentioned set of independent variables. For the comparison of 30-day mortality between the primary NIV and the primary IMV groups we performed two separate sensitivity analyses: (1) after exclusion of patients in whom LST limitation was not introduced during the initial respiratory treatment, and (2) after exclusion of patients in whom LST limitation was introduced within 30 days since the ICU admission.

This was a complete case analysis. Data missingness maps for each model are presented in Additional file 1: Figure S1. A two-sided *p*-value < 0.05 was considered statistically significant. Statistical analyses were performed using the R 4.1.0 software (R Development Core Team, Vienna, Austria).

## Results

### Characteristics of the study sample

**Fig. 1:**
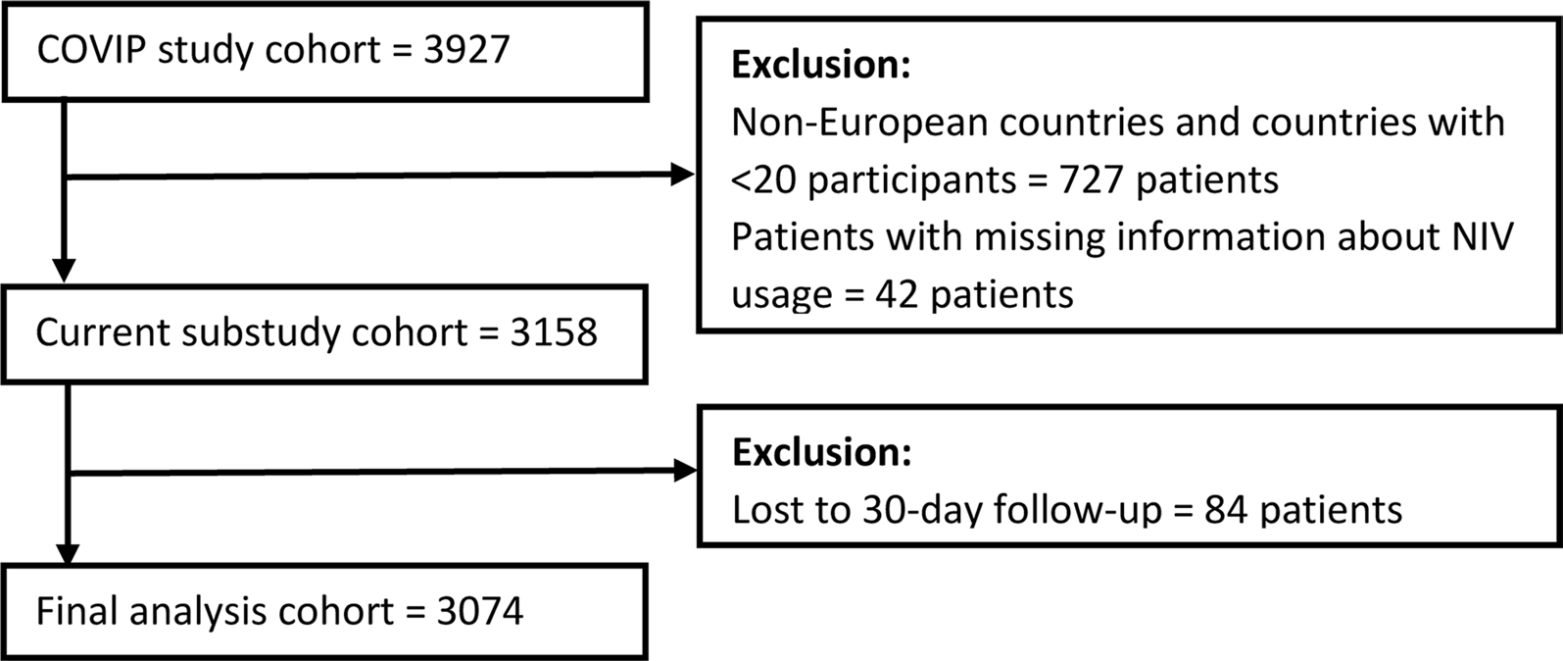
Study flowchart

The cohort of this COVIP substudy comprised 3158 critically ill patients recruited in 156 centres across 15 countries. All analyses were performed among patients with complete 30-day follow-up (3074/3158, 97.3%). The study flowchart is presented in Fig. 1. The mean age was 75.7 (SD 4.6) years, and most of the patients were male (2197/3074, 71.4%). The median SOFA score at admission was 5 (IQR 3–8). Based on the CFS score patients were classified as fit (67.0%), vulnerable (15.6%) and frail (17.4%). The baseline characteristics of the study group are presented in Table 2.

**Table 2:** Cohort characteristics

Characteristics	Entire cohort (n = 3074)	NIV (n = 791)	Primary NIV (n = 630)	Post-extubation NIV (n = 157)
Age, mean (SD) [years]	75.7 (4.6)	76.4 (4.9)	76.8 (4.9)	74.9 (4.6)
Female gender	877 (28.5)	226 (28.6)	181 (28.7)	42 (26.8)
BMI [kg/m^2^]	27.7 (24.8, 31.0)	27.7 (24.7, 31.3)	27.7 (24.5, 31.3)	27.8 (25.7, 31.5)
Prior hospitalization length [days]	2.0 (1.0, 5.0)	2.0 (1.0, 4.0)	2.0 (1.0, 5.0)	2.0 (1.0, 4.0)
Symptoms before hospitalization [days]	7.0 (4.0, 10.0)	7.0 (3.0, 10.0)	6.0 (3.0, 9.0)	7.0 (4.0, 10.0)
Diabetes	1033 (33.7)	293 (37.1)	241 (38.4)	51 (32.5)
Ischemic heart disease	694 (22.9)	190 (24.4)	158 (25.5)	31 (19.7)
Chronic renal failure	495 (16.2)	155 (19.7)	134 (21.4)	20 (12.7)
Arterial hypertension	2028 (66.2)	528 (67.0)	418 (66.7)	106 (67.5)
Pulmonary disease	689 (22.5)	199 (25.3)	159 (25.4)	40 (25.5)
Congestive heart failure	455 (15.0)	129 (16.5)	104 (16.8)	25 (15.9)
Bacterial coinfection	651 (21.6)	201 (26.1)	161 (26.4)	40 (25.6)
SOFA score on admission	5.0 (3.0, 8.0)	4.0 (3.0, 7.0)	4.0 (3.0, 6.0)	6.0 (4.0, 8.0)
Frailty status				
Fit (CFS 1–3)	1912 (67.0)	459 (61.2)	356 (59.6)	102 (68.0)
Vulnerable (CFS 4)	444 (15.6)	127 (16.9)	93 (15.6)	32 (21.3)
Frail (CFS 5–9)	498 (17.4)	164 (21.9)	148 (24.8)	16 (10.7)
Day of NIV initiation	1.0 (1.0, 3.0)	1.0 (1.0, 3.0)	1.0 (1.0, 1.0)	12.0 (8.0, 19.0)
Duration of NIV [hours]	34.0 (10.0, 88.0)	34.0 (10.0, 88.0)	34.0 (10.0, 91.3)	33.5 (12.8, 72.0)
IMV	2219 (72.2)	490 (61.9)	330 (52.4)	157 (100.0)
Day of IMV initiation	1.0 (1.0, 2.0)	2.0 (1.0, 3.0)	2.0 (2.0, 5.0)	1.0 (1.0, 1.0)
Vasopressors	2165 (70.8)	497 (63.4)	352 (56.3)	142 (91.6)
Renal replacement therapy	488 (15.9)	120 (15.2)	94 (14.9)	25 (15.9)
Antibiotics	2767 (90.1)	713 (90.1)	557 (88.4)	153 (97.5)
Steroids	2058 (68.6)	565 (74.3)	467 (77.3)	96 (63.2)
LST limitation	1189 (39.1)	306 (39.0)	276 (44.1)	28 (18.1)
Withholding	981 (32.3)	251 (32.1)	227 (36.3)	23 (14.8)
Withdrawal	627 (20.6)	161 (20.5)	150 (24.0)	9 (5.8)

*BMI*—body mass index, *CFS*—Clinical Frailty Scale, *IMV*—invasive mechanical ventilation, *LST*—life-sustaining therapy, *NIV*—noninvasive ventilation, *SOFA*—Sequential Organ Failure Assessment

### Noninvasive ventilation application across included countries

Noninvasive ventilation was used in 791 (25.7%) patients. NIV was used as a primary mechanical ventilation modality in 630 (79.6%) patients and as post-extubation respiratory support in 157 (19.8%) patients, while the day of NIV introduction was unknown in 4 patients (0.5%). The frequency of NIV use varied significantly across included countries and ranged from 1.1% (95% CI 0.1–4.1%) in Belgium to 62.0% (95% CI 47.2–75.4%) in Wales. NIV was used more commonly as primary therapy in all countries, but the distribution of NIV application differed depending on the country. We did not observe any evident temporal trend in the frequency of NIV in the study period (Additional file 1: Figure S2). Detailed information about the frequency and indications for NIV across participating countries is summarized in Table 1.

In the primary NIV group noninvasive ventilation was initiated on day 1.0 (IQR 1.0–1.0) and the median duration of NIV therapy was 33.5 h (12.75–72.0). The histograms of the day of NIV initiation and the duration of NIV therapy are presented in Additional file 1: Figure S3.

### Clinical outcomes

**Fig. 2:**
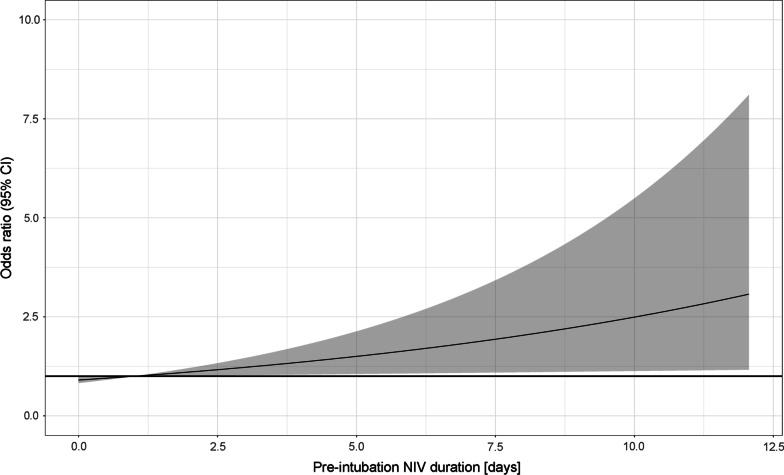
Association between pre-intubation NIV duration and 30-day mortality. Black line represents OR point estimate across NIV duration prior to endotracheal intubation, while grey areas depicts 95% confidence interval

Mortality at 30 days was 52.9% (333/630) in the primary NIV group and 14.0% (22/157) in the post-extubation NIV group. Among the 630 patients primarily treated with NIV, 330 (52.4%) patients eventually required invasive mechanical ventilation at a median 1.0 (0.0–3.0) days from the initiation of NIV. Among patients primarily treated with NIV, we did not find sufficient evidence for a difference in 30-day mortality between patients who eventually required endotracheal intubation and the remaining patients (58.2 vs. 47.0%, log-rank *p* = 0.32). The Kaplan–Meier curve for this comparison is presented in Additional file 1: Figure S4. The association between the duration of NIV and 30-day mortality in patients who eventually required intubation is visualized in Fig. 2.

### Factors associated with NIV failure

NIV failure, defined as endotracheal intubation or death within 30 days since ICU admission, occurred in 470/629 (74.7%) of patients primarily treated with NIV (date of intubation was unknown for one patient). Univariate comparison of these groups is summarized in Additional file 1: Table S3. A multivariable analysis revealed that higher SOFA score at admission (OR 3.73, 95% CI 2.36–5.90, *p* < 0.001) and higher baseline CFS (OR 1.46, 95% CI 1.06–2.00, *p* = 0.02) were associated with a higher risk for NIV failure, while hypertension was linked to lower odds of NIV failure (OR 0.50, 95% CI 0.31–0.81, *p* = 0.005). For a sensitivity analysis, we excluded patients in whom the decision to withhold or withdraw life-sustaining treatment was made within 2 days since admission to the ICU. This analysis revealed a NIV failure rate accounting to 74.1% (403/544) and showed its association with a higher SOFA score on admission (OR 3.38, 95% CI 2.10–5.46, *p* < 0.001) and hypertension (OR 0.58, 95% CI 0.35–0.96, *p* = 0.034). Finally, among patients in the primary NIV group in whom LST limitation was not introduced during the initial NIV treatment, the intubation rate was 67.4% (329/488) and was associated with the baseline SOFA score (OR 2.56, 95% CI 1.82–3.59, *p* < 0.001) and age (0.70, 95% CI 0.49–0.99, *p* = 0.044). Additional file 1: Table S4 presents the mortality, NIV failure rate, and intubation rate stratified by the duration of primary NIV.

### Primary NIV vs. primary IMV and 30-day mortality

**Fig. 3:**
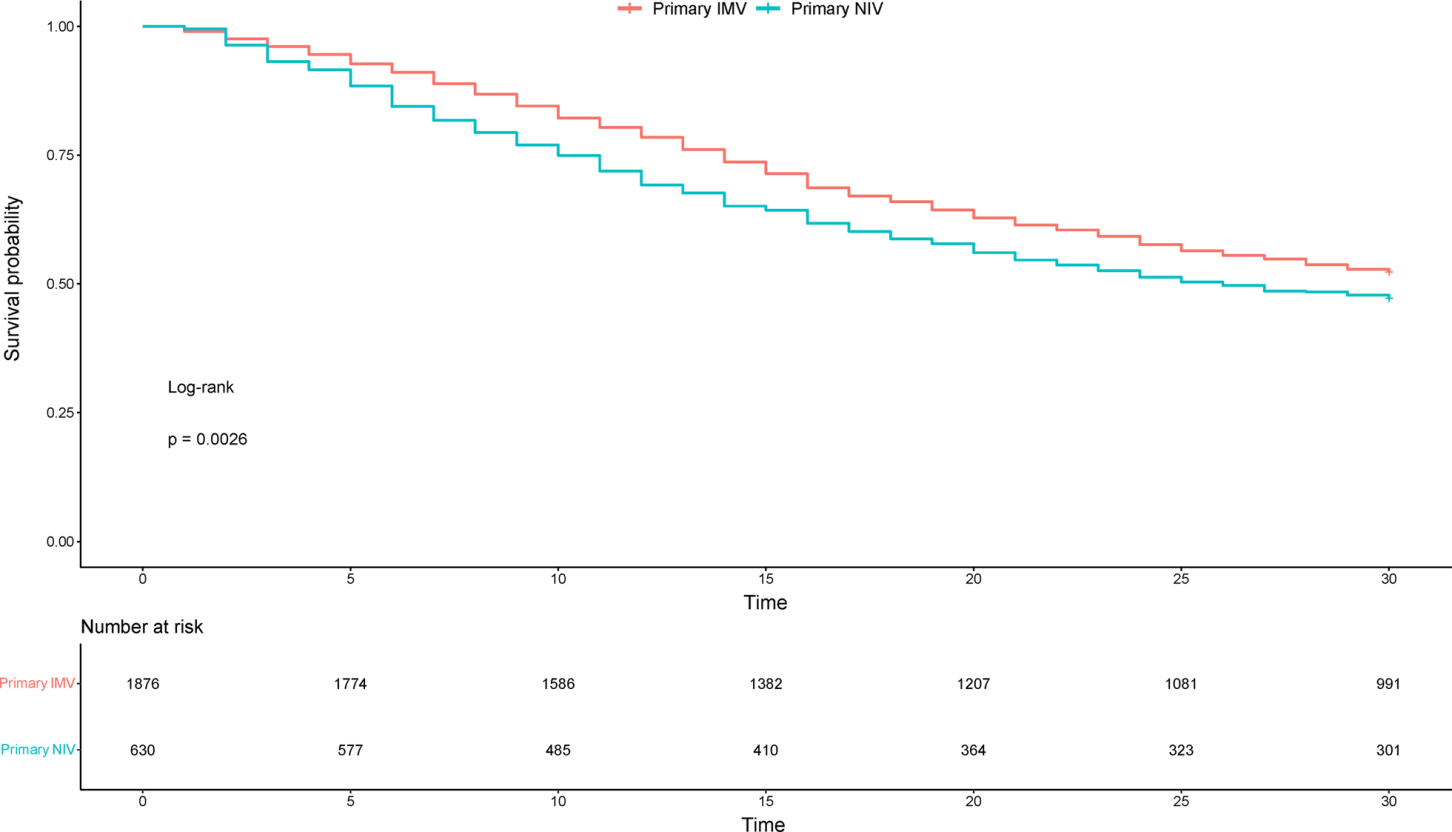
Kaplan–Meier curve for comparison of 30-day mortality between primary NIV and primary IMV group

Noninvasive ventilation and invasive ventilation were used as primary ventilation modality in 630 and 1876 patients, respectively. Compared to patients initially treated with IMV, patients in the primary NIV group were older (76.8 vs. 75.1, *p* < 0.001), more frequently frail (24.8 vs. 13.7%, *p* < 0.001), more commonly had diabetes (38.4 vs. 32.5%, *p* = 0.009), IHD (25.5 vs. 20.5%, 0.01), chronic renal failure (21.4 vs. 13.3%, *p* < 0.001), chronic pulmonary disease (25.4 vs. 21.4%, *p* = 0.044), congestive heart failure (16.8 vs. 13.3%, *p* = 0.034) and had a lower SOFA score on admission (4 vs 7, *p* < 0.001). Life-sustaining treatment was withheld more commonly in the primary NIV group (36.3 vs. 29.5%, *p* = 0.002) and withdrawn similarly often in both groups (24.0 vs. 22.5%, *p* = 0.495). The distribution of LST limitation timing is presented in Additional file 1: Figure S5. Detailed univariate comparison of the groups is presented in Additional file 1: Table S5. We found a significantly lower crude 30-day mortality in the primary IMV group compared to the primary NIV group (47.7 vs. 52.9%, log-rank *p* = 0.003) (Fig. 3). A multivariable restricted mean survival time analysis revealed that in the 30-day follow-up patients in the primary NIV group lived for 1.36 fewer days (95% CI − 2.27 to − 0.46 days, *p* = 0.003) compared to the patients in the primary IMV group.

We performed two sensitivity analyses to account for LST limitation in these groups. The first sensitivity analysis revealed that, after exclusion of patients in whom LST limitation was introduced during the initial respiratory treatment (i.e., during NIV in the primary NIV group and during IMV in the primary IMV group), 30-day mortality in the primary NIV was higher than in the primary IMV group (43.2 vs. 29.5%, log-rank *p* < 0.001). For the second sensitivity analysis, we excluded all patients in whom LST limitation was introduced within 30 days since the ICU admission. It suggested a lack of association between the modality of the initial respiratory support and 30-day mortality (28.0 vs. 29.5%, log-rank *p* < 0.55). The Kaplan–Meier curves for these comparisons are presented in Additional file 1: Figure S6.

## Discussion

This prospective observational study of more than 3000 patients aged ≥ 70 years old hospitalized in the ICU due to severe COVID-19 showed that the use of NIV in this population is highly variable in Europe and is associated with high failure and mortality rates. A longer time to intubation in patients requiring escalation from NIV to IMV was associated with an increased 30-day mortality. Finally, a comparison of initial respiratory support strategies revealed that primary IMV was associated with lower mortality compared to primary NIV.

At the moment, NIV is strongly recommended by the combined European Respiratory Society and American Thoracic Society task force in two clinical scenarios, i.e., hypercapnic ARF due to COPD exacerbation and cardiogenic pulmonary oedema [18]. Previous reports by the VIP Study Group, based on a cohort of patients aged ≥ 80 years admitted to the ICUs before the pandemic, showed that every fourth patient received NIV [19]. In the light of a universal shortage of ICU beds during the COVID-19 pandemic, NIV became a promising alternative to IMV in patients with severe hypoxemic ARF. Such approach was also justified by encouraging results of a recent Bayesian network analysis [12]. The current study showed that NIV was used in approximately a quarter of patients included in this study; however, the frequency of NIV application was highly variable across the Europe. This is in line with previous reports revealing significant international variation in management of ARF in the course of COVID-19 [20]. The presented inter-country differences are multifactorial and are probably related to local availability of ICU beds, presence of intermediate care units, and national management strategies. The clinical relevance of noninvasive respiratory strategies increased significantly during COVID-19 due to a dramatic increase in the number of hypoxemic ARF cases. On the one hand, the main idea behind the implementation of NIV in this clinical scenario was to avoid intubation and admission to the ICU. On the other hand, the high failure rate raised concerns that NIV may only delay endotracheal intubation and potentially worsen patients outcomes [21, 22]. Other potentially alarming aspects of NIV application in patients with hypoxemic ARF due to COVID-19 included the risk of self-inflicted lung injury secondary to large tidal volumes as well as the generation of aerosol increasing the risk of nosocomial infections [23]. Our study confirmed that more than half of patients initially treated with NIV eventually required endotracheal intubation and NIV failure, defined as intubation or death within 30 days since the NIV initiation, was observed in almost 75% of the cases. In comparison, the primary outcome, including intubation or death in the continuous positive airway pressure (CPAP) group of the RECOVERY RS trial, occurred in 36.3% of the patients, and 28-day mortality and intubation rate in the helmet NIV group of the HENIVOT trial were 15% and 30%, respectively [15, 16]. This discrepancy may be largely attributed to older age, a higher number of comorbidities, more frequent coexistence of frailty, and greater severity of the disease expressed as a higher SOFA score at admission in the COVIP study sample. Nevertheless, such a high NIV failure rate was expected since elderly ICU patients have a particularly grim prognosis [24]. Therefore, in order to avoid unnecessary invasive procedures and futile suffering of the patients, LST limitations are commonly introduced during hospitalization. In our study, any form of LST limitation (withhold or withdrawal) was applied at some point of hospitalization in more than 40% of cases. This proportion is markedly higher compared to a cohort of merged VIP1 and VIP2 studies in which therapy limitation was introduced in 32% of patients [19, 25]. We believe that this difference is yet another consequence of the ICUs being overflown by a staggering number of critically ill patients during the ongoing pandemic [26]. As a result, more advanced treatment modalities such as IMV and extracorporeal membrane oxygenation became reserved for younger and healthier patients with a more favourable prognosis.

Despite years of research, the risk factors for NIV failure remain unclear. Most of the evidence on this issue is based on low-quality studies concerning predominantly hypercapnic ARF [27–29]. Some interesting insights were offered by a prospective observational study including over 1800 patients with ARF due to influenza, which suggested an increasing SOFA score as a predictor of NIV failure [30]. In our study increasing baseline SOFA score was related to higher risk of NIV failure as well as higher intubation rate in patients without LST limitation during the primary NIV therapy. Additionally, we revealed an association between an increasing degree of frailty and the risk of NIV failure. This is consistent with previous studies describing the impact of frailty on outcomes in elderly patients admitted to the ICU [14, 19].

It is crucial for a clinician not to cause harm while trying to avoid intubation by using NIV. In general, the use of NIV is restricted to mild ARDS with a success rate of 78%, decreasing to 58% and 53% for moderate and severe ARDS [21]. Our analysis of the association between the duration of NIV before intubation and 30-day mortality revealed a poorer prognosis in a subgroup of patients in whom NIV duration exceeded 3 days. It suggests that clinicians should reevaluate patients in terms of indications for intubation early in the course of NIV therapy when there are no signs of improvement. This could potentially prevent excessive delays in intubation and therefore improve patients’ outcomes.

Taking into account the high failure rate and the well-established relation between delays in intubation and a poorer prognosis, we compared the outcomes of patients depending on an initially introduced modality of respiratory support. Survival analysis revealed that mortality was higher in patients treated primarily with NIV. Similar observation was made in a sensitivity analysis that excluded patients in whom LST was limited during NIV in the primary NIV group and during IMV in the primary IMV group. However, the difference in mortality between the groups disappeared after the exclusion of all patients in whom LST limitation was introduced within 30 days since the ICU admission. Another interesting observation is similar mortality in the primary IMV group and in patients who were treated with NIV and never required intubation (47.7% and 47.0%, respectively) and markedly higher mortality in patients who required intubation after the initial NIV trial (58.2%). On the one hand, these results are not very surprising because NIV is a suboptimal therapy for the majority of ARDS cases according to the current guidelines and its application in this clinical scenario is a last resort for elderly patients who would likely not be qualified for intubation under pandemic circumstances. On the other hand, the results of the second sensitivity analysis suggest that both primary NIV and primary IMV may be associated with a similar survival rate in elderly critically ill COVID-19 patients when LST limitation is not considered.

The main strengths of this paper include an international multicentre character of the study and a relatively large sample of a very specific population. Moreover, the small amount of missing data on 30-day mortality increases the credibility of our findings. We are aware of several limitations of this article. First, some details concerning NIV technique were not gathered i.e., type of interface (face mask vs. helmet) and mode of ventilation (CPAP vs. bi-level). Second, we did not gather data on HFNOT, an option increasingly used in patients with respiratory failure before tracheal intubation and IMV. Third, participating countries are probably largely heterogenous in terms of intubation criteria, which likely affects our results in a significant way. Fourth, we did not collect any physiological or clinical data describing the initial response of patients to NIV therapy, which could potentially provide very valuable information, particularly in terms of risk factors for NIV failure. Finally, potentially important data on the pre-ICU disease trajectory was not collected. Therefore, we were unable to determine whether and for how long NIV or HFNOT were used before admission to the ICU.

## Conclusions

In conclusion, NIV is used in approximately one in four elderly patients with COVID-19 treated in the European ICU, and its use varies significantly across the European countries. Initial application of NIV was associated with a high risk of failure and increased mortality compared to patients in whom IMV was the first mechanical ventilation modality. In addition, we found evidence of the association between higher SOFA and CFS scores and an increased risk of NIV failure. Finally, among patients primarily treated with NIV who eventually required IMV, delay in endotracheal intubation was associated with increased 30-day mortality. Careful monitoring during the first days of NIV is essential to ensure that patients showing no sign of improvement from NIV, who are expected to benefit from an escalation to IMV, are offered endotracheal intubation promptly.

## Supplementary Information

**Additional file 1.** **Supplementary Figure 1.** Missingness maps for each model included in the paper. **Supplementary Figure 2.** Histograms showing (A) number of recruited patients (B) proportion of primary NIV (C) proportion of primary IMV and (D) 30-day mortality stratified by study month. **Supplementary Figure 3.** Histograms showing distribution of NIV initiation day and NIV duration stratified by group. **Suuplementary Figure 4.** Kaplan-Meier curves comparing survival in patients with primary NIV stratified by intubation status. **Supplementary Figure 5.** Distribution of LST withhold and withdrawal timing. **Supplementary Figure 6.** Kaplan-Meier curves for the sensitivity analyses. **Supplementary Table 1.** Details concerning ethical approval and patient`s consent requirements in participating countries. **Supplementary Table 2.** Definitions of comorbidities. **Supplementary Table 3.** Comparison of patients primarily treated with NIV stratified by NIV failure. **Supplementary Table 4.** 30-day mortality, NIV failure rate and intubation rate stratified by the duration of primary NIV. **Supplementary Table 5.** Comparison of patients primarily treated with NIV and IMV.

## Data Availability

The datasets used and/or analysed during the current study are available from the corresponding author on reasonable request.

## Abbreviations

ARDS: Acute respiratory distress syndrome
ARF: Acute respiratory failure
CFS: Clinical Frailty Scale
COVID-19: Coronavirus disease 2019
HFNOT: High-flow nasal oxygen therapy
ICU: Intensive care unit
IHD: Ischaemic heart disease
IMV: Invasive mechanical ventilation
NIV: Noninvasive ventilation
IQR: Interquartile ranges
LST: Life-sustaining therapies
RT-PCR: Reverse transcription polymerase chain reaction
SOFA: Sequential organ failure assessment
VIP: Very old intensive care patients
