# Supplementary material for: Noninvasive ventilation in COVID-19 patients aged ≥ 70 years—a prospective multicentre cohort study
Source: Crit Care. 2022 Jul 22;26:224. doi: 10.1186/s13054-022-04082-1 (PMC9305028; doi:10.1186/s13054-022-04082-1)
Supplement: Supplementary file 1 — Additional file 1. Supplementary Figure 1. Missingness maps for each model included in the paper. Supplementary Figure 2. Histograms showing (A) number of recruited patients (B) proportion of primary NIV (C) proportion of primary IMV and (D) 30-day mortality stratified by study month. Supplementary Figure 3. Histograms showing distribution of NIV initiation day and NIV duration stratified by group. Suuplementary Figure 4. Kaplan-Meier curves comparing survival in patients with primary NIV stratified by intubation status. Supplementary Figure 5. Distribution of LST withhold and withdrawal timing. Supplementary Figure 6. Kaplan-Meier curves for the sensitivity analyses. Supplementary Table 1. Details concerning ethical approval and patient`s consent requirements in participating countries. Supplementary Table 2. Definitions of comorbidities. Supplementary Table 3. Comparison of patients primarily treated with NIV stratified by NIV failure. Supplementary Table 4. 30-day mortality, NIV failure rate and intubation rate stratified by the duration of primary NIV. Supplementary Table 5. Comparison of patients primarily treated with NIV and IMV. [file 13054_2022_4082_MOESM1_ESM.docx]

**SUPPLEMENTARY MATERIAL**

**Title:** Non-invasive ventilation in COVID-19 patients aged ≥70 years – a prospective multicentre cohort study.

**Authors:** Kamil Polok, Jakub Fronczek, Antonio Artigas, Hans Flaatten, Bertrand Guidet, Dylan W. De Lange, Jesper Fjølner, Susannah Leaver, Michael Beil, Sigal Sviri, Raphael Romano Bruno, Bernhard Wernly, Bernardo Bollen Pinto, Joerg C. Schefold, Dorota Studzińska, Michael Joannidis, Sandra Oeyen, Brian Marsh, Finn H. Andersen, Rui Moreno, Maurizio Cecconi, Christian Jung, Wojciech Szczeklik on behalf of COVIP study group

**List of content:**

**Supplementary Figure 1.** Missingness maps for each model included in the paper.

**Supplementary Figure 2.** Histograms showing (A) number of recruited patients (B) proportion of primary NIV (C) proportion of primary IMV and (D) 30-day mortality stratified by study month.

**Supplementary Figure 3.** Histograms showing distribution of NIV initiation day and NIV duration stratified by group.

**Suuplementary Figure 4**. Kaplan-Meier curves comparing survival in patients with primary NIV stratified by intubation status

**Supplementary Figure 5.** Distribution of LST withhold and withdrawal timing.

**Supplementary Figure 6.** Kaplan-Meier curves for the sensitivity analyses.

**Supplementary Table 1.** Details concerning ethical approval and patient`s consent

requirements in participating countries.

**Supplementary Table 2.** Definitions of comorbidities

**Supplementary Table 3.** Comparison of patients primarily treated with NIV stratified by NIV failure.

**Supplementary Table 4.** 30-day mortality, NIV failure rate and intubation rate stratified by the duration of primary NIV.

**Supplementary Table 5.** Comparison of patients primarily treated with NIV and IMV.

**Supplementary Figure 1.** Missingness maps for each model included in the paper.


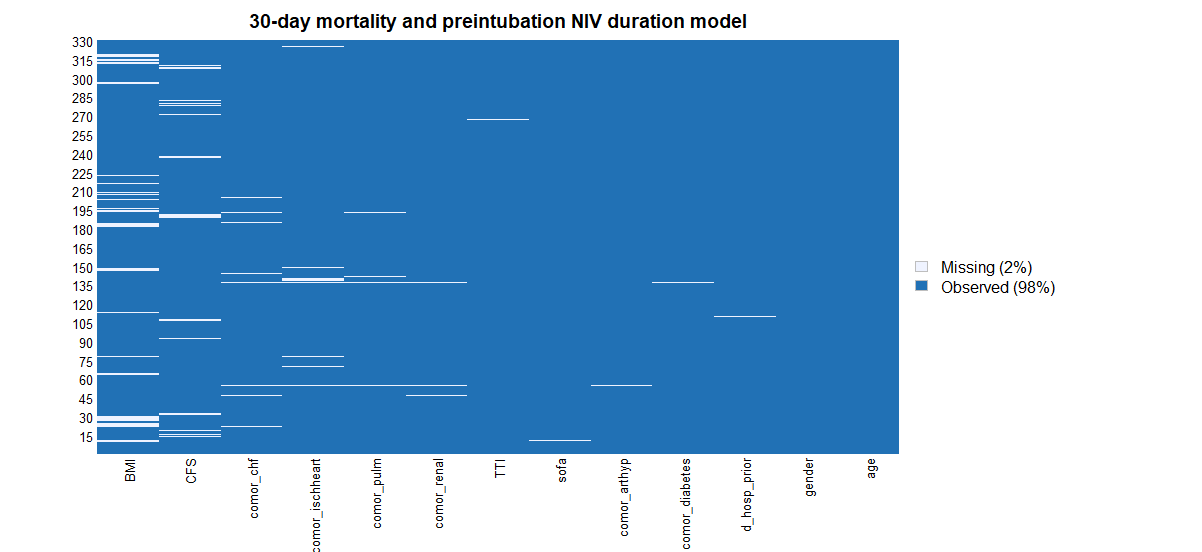


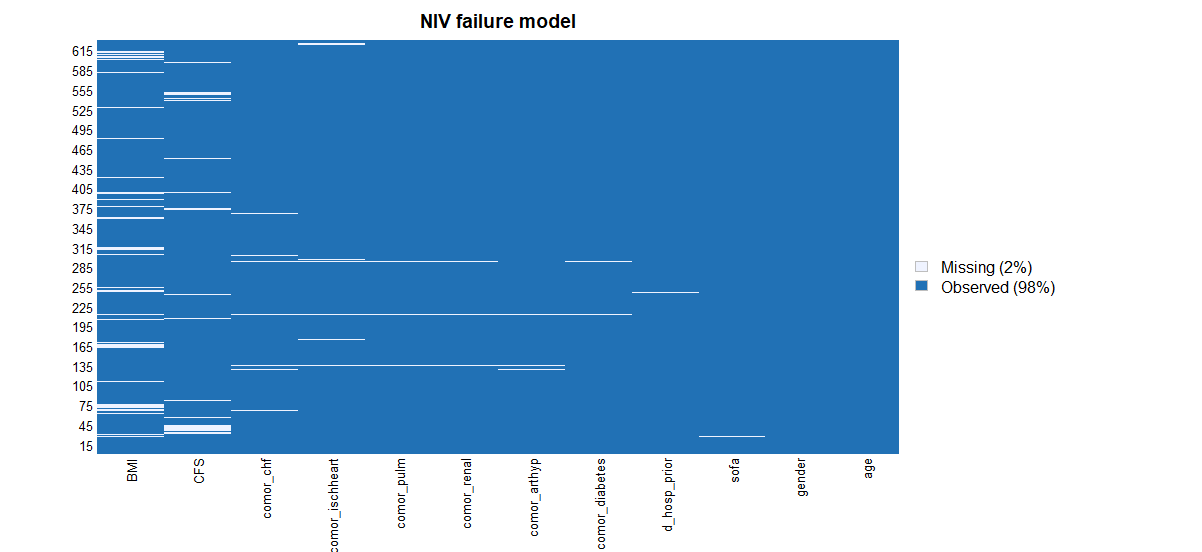


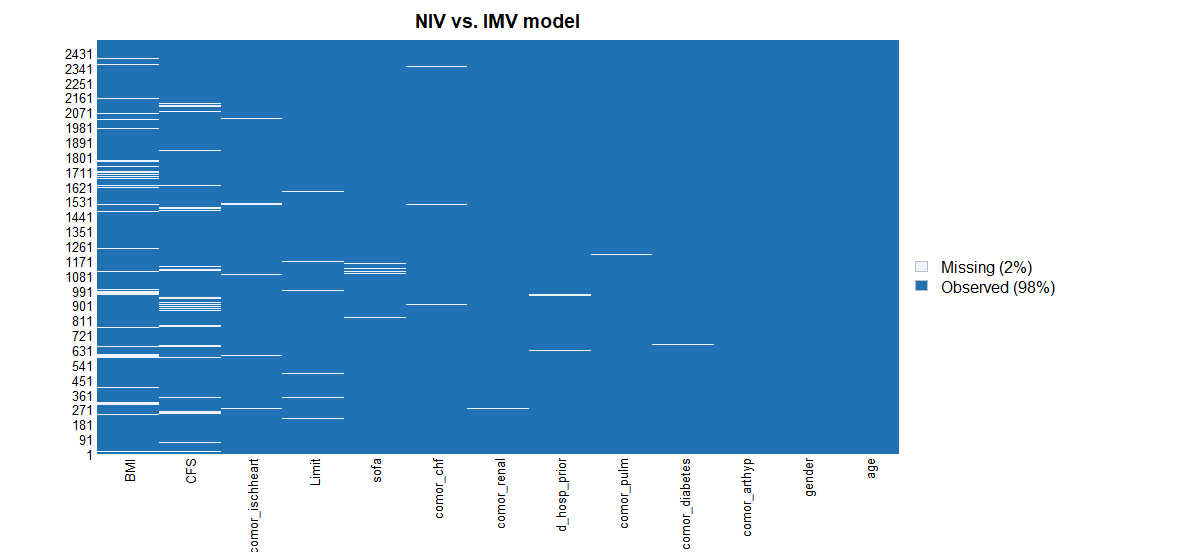


**Supplementary Figure 2.** Histograms showing (A) number of recruited patients (B) proportion of primary NIV (C) proportion of primary IMV and (D) 30-day mortality stratified by study month.


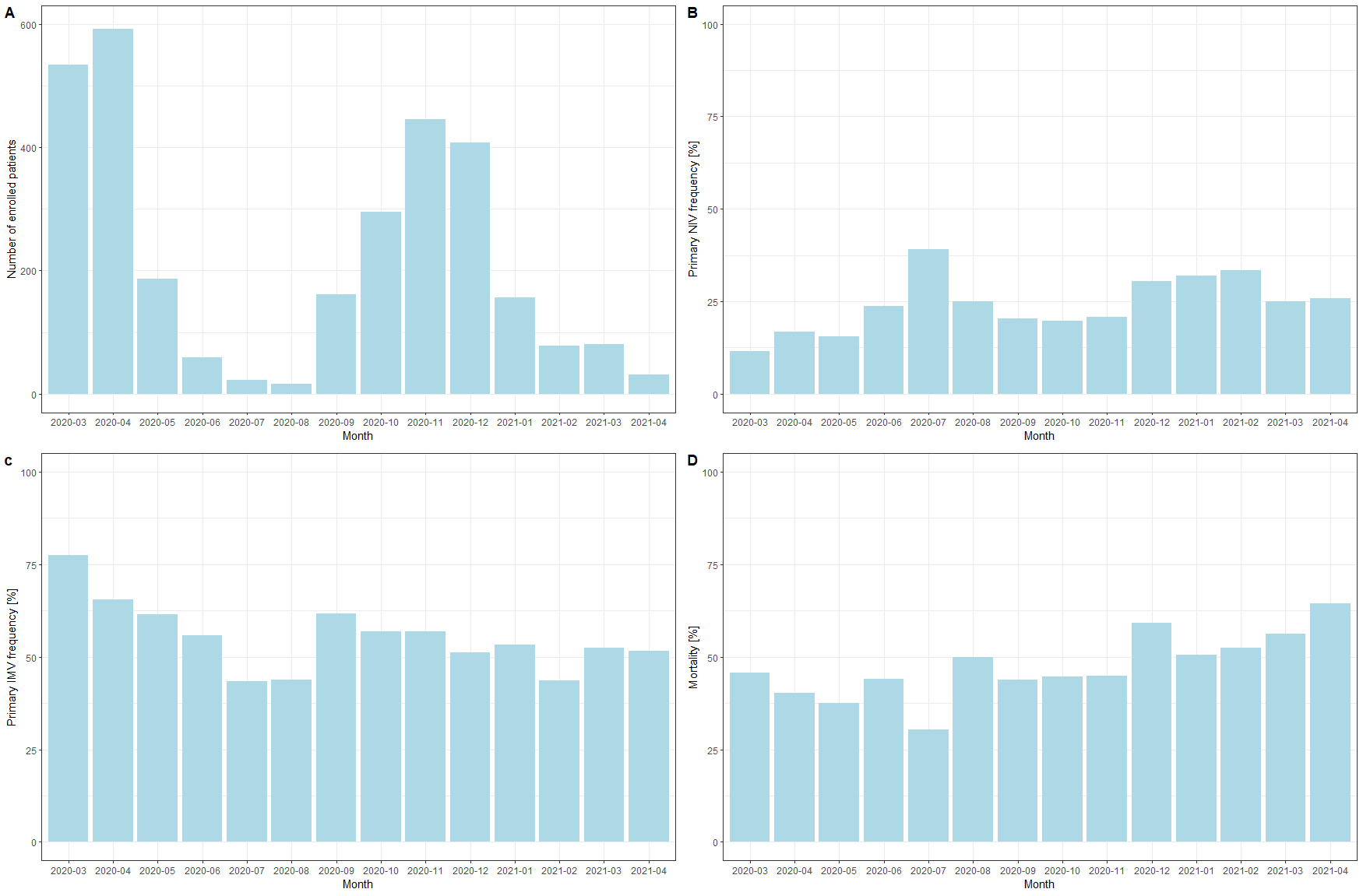


**Supplementary Figure 3.** Histograms showing distribution of NIV initiation day and NIV duration stratified by group.


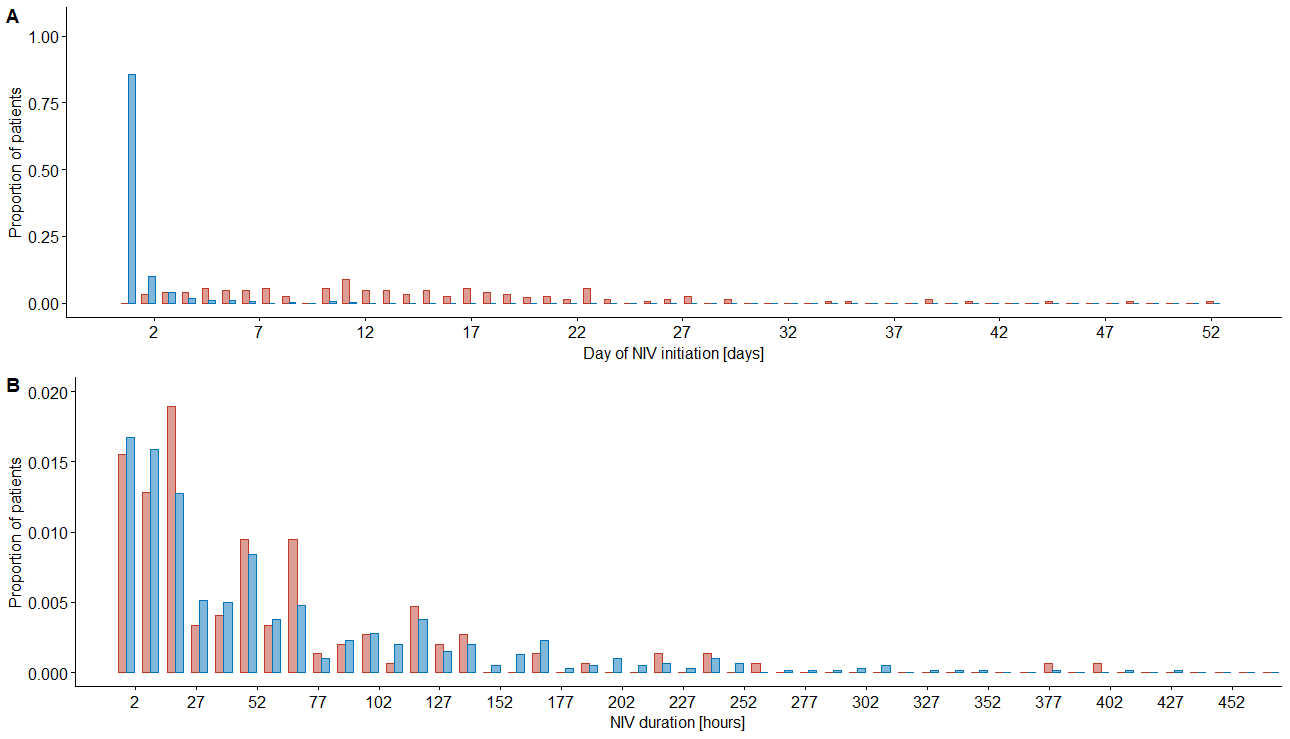


**Footnote:** Primary NIV is depicted in blue while Post-extubation NIV is presented with pink.

**Suplementary Figure 4**. Kaplan-Meier curves comparing survival in patients with primary NIV stratified by IMV status.


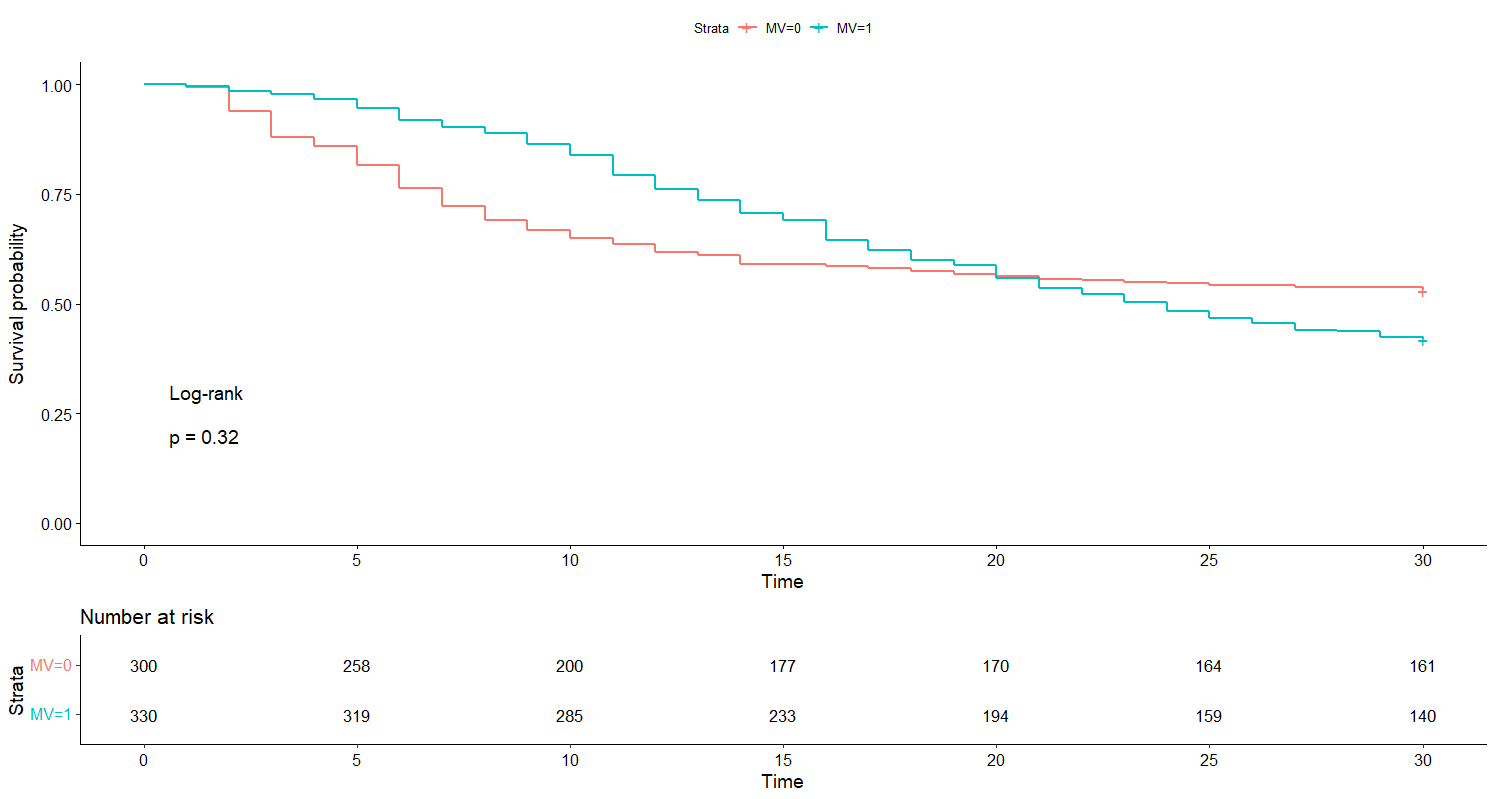


**Supplementary Figure 5.** Distribution of LST withhold and withdrawal timing.


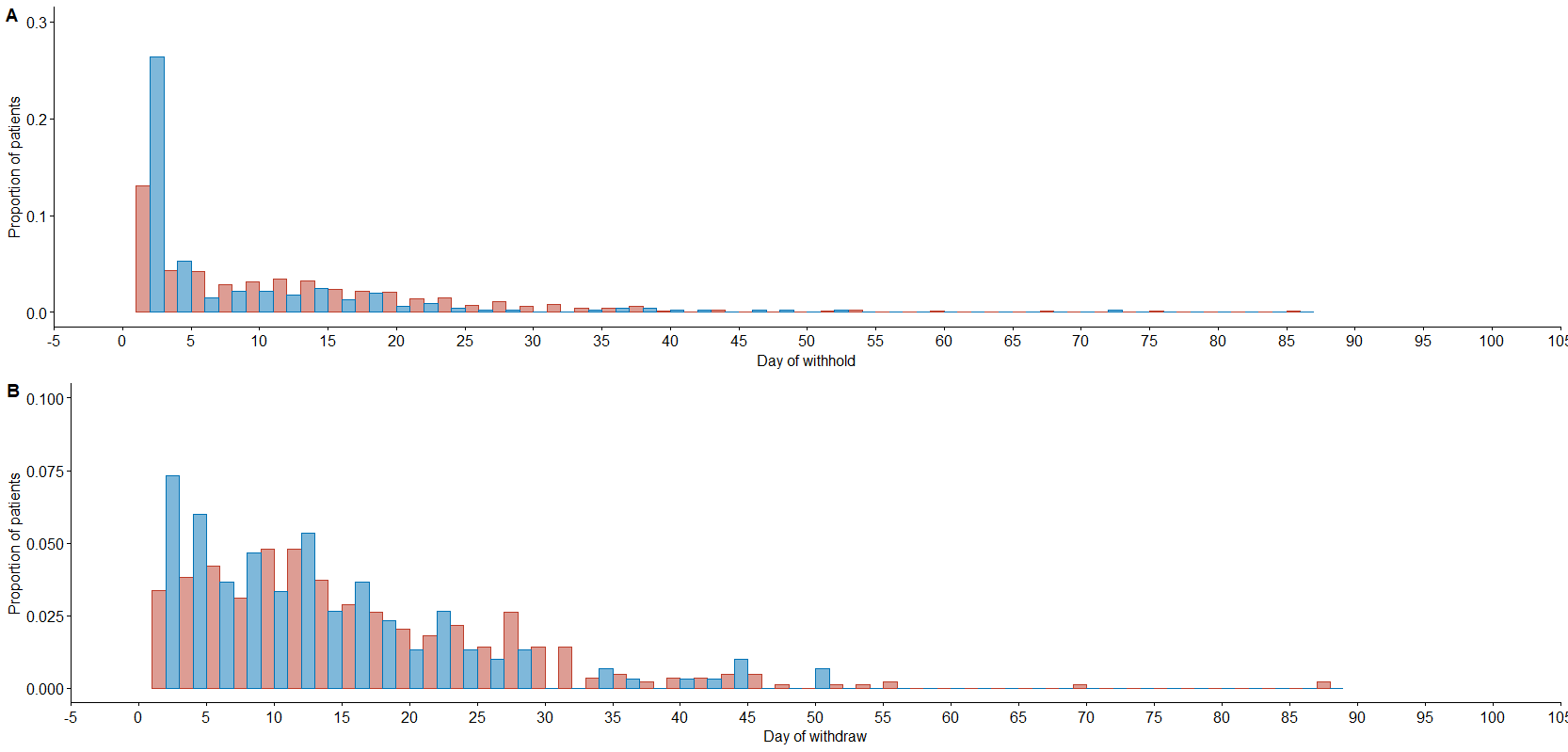


**Footnote:** Primary NIV is depicted in blue while primary IMV is presented with pink.

**Supplementary Figure 6.** Kaplan-Meier curves for the sensitivity analyses.


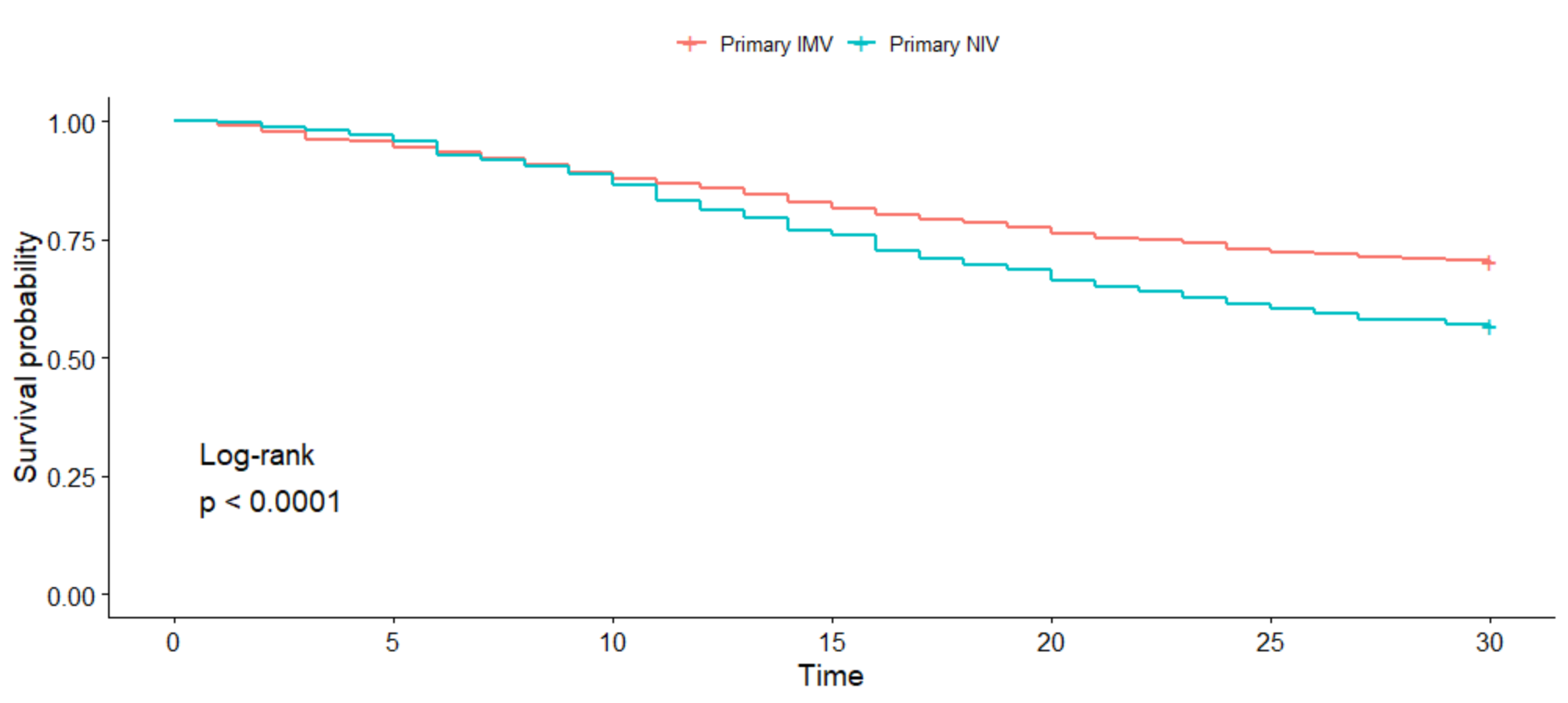


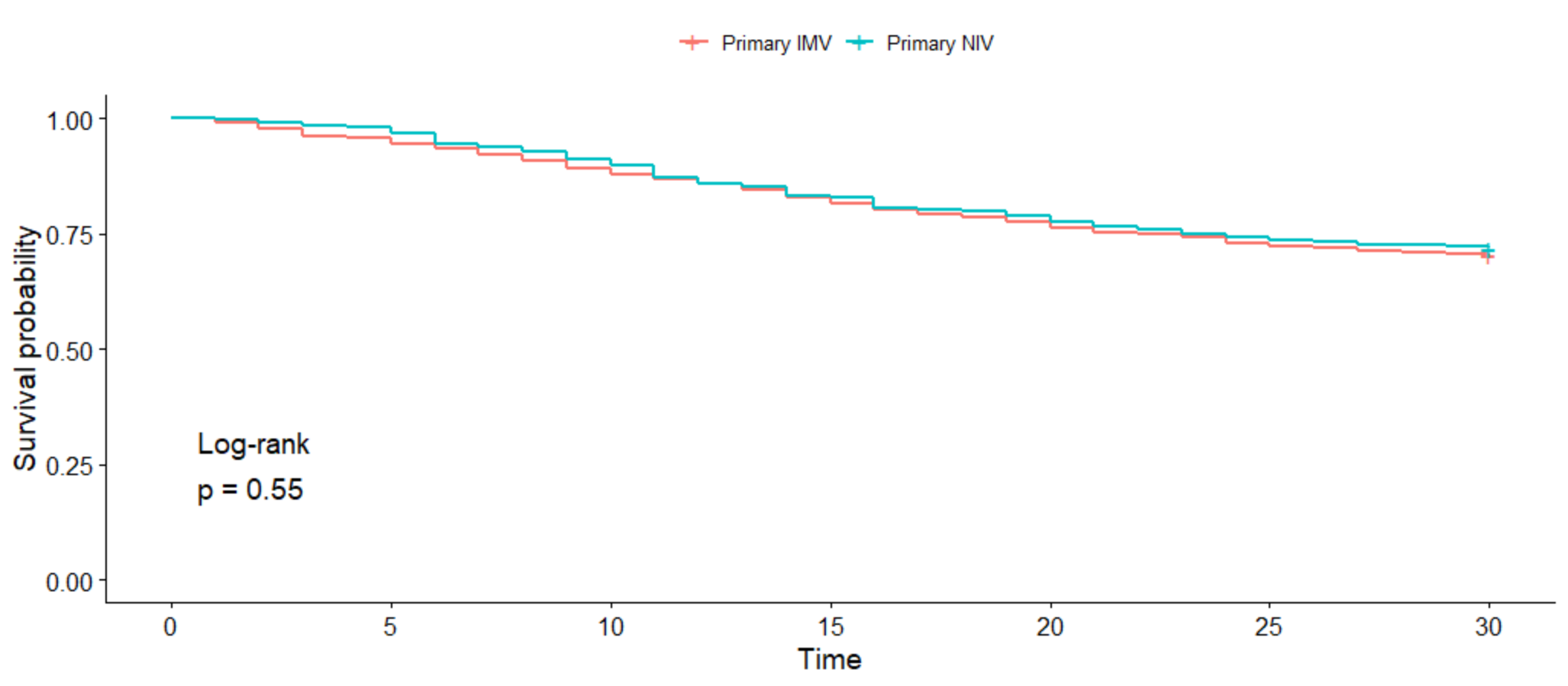


**Footnote:** The first curve compares the groups after exclusion of patients in whom LST limitation was not introduced during the initial respiratory treatment. The latter presents the comparision of survival probabilty after exclusion of patients in whom LST limitation was introduced within 30 days since the ICU admission.

**Supplementary Table 1.** Details concerning ethical approval and patient`s consent

requirements in participating countries.

| **Country** | **Details on ethical board approval** | **Details on patient's consent** |
| --- | --- | --- |
| Austria | Ethical approval was acquired nationally and for each individual hospital. | Required |
| Belgium | Ethical approval was acquired nationally and for each individual hospital. | Required |
| Denmark | Ethical approval was acquired nationally. | Waived |
| England | Ethical approval was acquired nationally and for each individual hospital. | Waived |
| France | Ethical approval was acquired nationally | Required |
| Germany | Ethical approval was acquired nationally and for each individual hospital. | Required |
| Greece | Ethical approval was acquired nationally and for each individual hospital. | Required |
| Ireland | Ethical approval was acquired for each individual hospital. | Deferred consent |
| Israel | Ethical approval was acquired for each individual hospital. | Waived |
| Netherlands | Ethical approval was acquired nationally and for each individual hospital. | Required |
| Norway | Ethical approval was acquired nationally | Waived. Deferred consent in survivors. |
| Poland | Ethical approval was acquired for each individual hospital. | Waived |
| Portugal | Ethical approval was acquired for each individual hospital. | Required in majority of centres |
| Spain | Ethical approval was acquired for each individual hospital. | Required in majority of centres |
| Switzerland | Ethical approval was acquired nationally and for each individual hospital. | Waived. Deferred consent at 3-month follow-up in survivors. |
| Wales | Ethical approval was acquired nationally and for each individual hospital. | Waived |

**Supplementary Table 2.** Definitions of comorbidities

| **Diabetes mellitus** | Documented evidence of diabetes mellitus before Hospital admission, reported by the patient or their relatives or prescription of anti-diabetic medication or insulin on the drug chart. |
| --- | --- |
| **Ischaemic heart disease** | Documented abnormal coronary angiography, known coronary artery disease, previous percutaneous coronary intervention (PCI) or coronary bypass surgery |
| **Chronic renal failure** | Documented evidence of chronic renal insufficiency Grade 3 or higher, creatinine clearance <60ml/min or chronic dialysis |
| **Arterial hypertension** | Documented evidence of any grade of chronic arterial hypertension and/or prescription of anti-hypertensive medication |
| **Pulmonary disease** | Documented evidence of or medication prescribed for chronic pulmonary disease of any aetiology (bronchial asthma, COPD, pulmonary fibrosis), or clinical or radiological signs of chronic pulmonary disease |
| **Chronic heart failure** | Documented evidence of or medication prescribed for chronic heart failure of any aetiology or echocardiographic or radiological signs of chronic heart failure. |

**Supplementary Table 3.** Comparison of patients primarily treated with NIV stratified by NIV failure.

| **Characteristics** | **NIV success (n=159)** | **NIV failure  (n=470)** | **p-value** |
| --- | --- | --- | --- |
| Age, mean (SD) [years] | 76.5 (4.8) | 76.9 (5.0) | 0.351 |
| Female gender | 52 (32.7) | 129 (27.4) | 0.244 |
| BMI [kg/m^2^] | 27.7 (24.6, 32.0) | 27.5 (24.5, 31.1) | 0.496 |
| Prior hospitalization length [days] | 2.0 (1.0, 4.0) | 2.0 (1.0, 5.0) | 0.397 |
| Duration of symptoms before hospitalization [days] | 7.0 (3.0, 10.0) | 6.0 (3.0, 9.0) | 0.628 |
| Diabetes | 60 (37.7) | 181 (38.7) | 0.908 |
| Ischemic heart disease | 36 (22.9) | 122 (26.5) | 0.441 |
| Chronic renal failure | 29 (18.2) | 105 (22.5) | 0.304 |
| Arterial hypertension | 115 (72.8) | 303 (64.7) | 0.079 |
| Pulmonary disease | 41 (25.8) | 118 (25.4) | 1.000 |
| Congestive heart failure | 25 (15.8) | 79 (17.2) | 0.788 |
| Bacterial coinfection | 28 (17.9) | 133 (29.4) | 0.007 |
| SOFA score on admission | 3.0 (2.0, 5.0) | 4.0 (3.0, 7.0) | <0.001 |
| Frailty status |  |  | 0.107 |
| Fit | 99 (66.4) | 257 (57.5) |  |
| Frail | 28 (18.8) | 120 (26.8) |  |
| Vulnerable | 22 (14.8) | 70 (15.7) |  |
| CFS score on admission | 3.0 (2.0, 4.0) | 3.0 (3.0, 5.0) | 0.005 |
| Day of NIV initiation | 1.0 (1.0, 1.0) | 1.0 (1.0, 1.0) | 0.140 |
| Duration of NIV [hours] | 48.0 (17.0, 106.0) | 30.0 (8.0, 82.0) | 0.004 |
| Vasopressors | 17 (10.8) | 334 (71.5) | <0.001 |
| Renal replacement therapy | 2 (1.3) | 92 (19.6) | <0.001 |
| Antibiotics | 123 (77.4) | 433 (92.1) | <0.001 |
| Steroids | 106 (70.7) | 360 (79.5) | 0.034 |

**Supplementary Table 4.** 30-day mortality, NIV failure rate and intubation rate stratified by the duration of primary NIV.

| Comparison | NIV duration | | | p-value |
| --- | --- | --- | --- | --- |
|  | <24 hours | 24-72 hours | >72 hours |  |
| **Primary NIV group (NIV failure = death or intubation within 30 days)** | | | | |
| 30-mortality | 127/240 (52.9) | 92/180 (51.1) | 106/184 (57.6) | 0.61 |
| NIV failure | 191/239 (79.9) | 132/180 (73.3) | 134/184 (72.8) | 0.16 |
| **Primary NIV group without LST limitation** | | | | |
| 30-mortality | 93/198 (47.0) | 62/145 (42.8) | 51/123 (41.5) | 0.39 |
| Intubation | 153/198 (77.3) | 94/145 (64.8) | 72/123 (58.5) | 0.001 |

**Footnote:** Data are presented as n/N (%)

**Supplementary Table 5.** Comparison of patients primarily treated with NIV and IMV.

| **Characteristics** | **Primary IMV**  **(n=1876)** | **Primary NIV**  **(n=630)** | **p-value** |
| --- | --- | --- | --- |
| Age, mean (SD) [years] | 75.1 (4.2) | 76.8 (4.9) | <0.001 |
| Female gender | 517 (27.6) | 181 (28.7) | 0.606 |
| BMI [kg/m2] | 27.7 (25.0, 30.9) | 27.65 (24.5, 31.3) | 0.899 |
| Prior hospitalization length [days] | 2.0 (1.0, 5.0) | 2.0 (1.0, 5.0) | 0.390 |
| Duration of symptoms before hospitalization [days] | 7.0 (4.0, 10.0) | 6.0 (3.0, 9.0) | 0.003 |
| Diabetes | 609 (32.5) | 241 (38.4) | 0.009 |
| Ischemic heart disease | 380 (20.5) | 158 (25.5) | 0.010 |
| Chronic renal failure | 249 (13.3) | 134 (21.4) | <0.001 |
| Arterial hypertension | 1229 (65.6) | 418 (66.7) | 0.655 |
| Pulmonary disease | 401 (21.4) | 159 (25.4) | 0.044 |
| Congestive heart failure | 247 (13.3) | 104 (16.8) | 0.034 |
| Bacterial coinfection | 414 (22.5) | 161 (26.4) | 0.053 |
| SOFA score on admission | 7.00 (4.00, 8.00) | 4.00 (3.00, 6.00) | <0.001 |
| Frailty status |  |  | <0.001 |
| Fit | 1222 (71.1) | 356 (59.6) |  |
| Frail | 235 (13.7) | 148 (24.8) |  |
| Vulnerable | 262 (15.2) | 93 (15.6) |  |
| LST limitation | 699 (37.7) | 276 (44.1) | 0.005 |
| Withholding | 547 (29.5) | 227 (36.3) | 0.002 |
| Withdrawal | 417 (22.5) | 150 (24.0) | 0.495 |
| Vasopressors | 1736 (93.0) | 352 (56.3) | <0.001 |
| Renal replacement therapy | 371 (19.8) | 94 (14.9) | 0.007 |
| Antibiotics | 1789 (95.5) | 557 (88.4) | <0.001 |
| Steroids | 1239 (67.6) | 467 (77.3) | <0.001 |
